# Short Communication: Reduced Nevirapine Concentrations Among HIV-Positive Women Receiving Mefloquine for Intermittent Preventive Treatment for Malaria Control During Pregnancy

**DOI:** 10.1089/aid.2018.0042

**Published:** 2018-11-01

**Authors:** Richard E. Haaland, Kephas Otieno, Amy Martin, Abraham Katana, Chuong Dinh, Laurence Slutsker, Clara Menendez, Raquel Gonzalez, John Williamson, Walid Heneine, Meghna Desai

**Affiliations:** ^1^ Division of HIV/AIDS Prevention, National Center for HIV/AIDS, Viral Hepatitis, STD, and TB Prevention, Centers for Disease Control and Prevention, Atlanta, Georgia.; ^2^ Kenya Medical Research Institute (KEMRI), Center for Global Health Research, Kisumu, Kenya.; ^3^ Malaria Branch, Division of Parasitic Diseases and Malaria, Center for Global Health, Centers for Disease Control and Prevention, Atlanta, Georgia.; ^4^ ISGlobal, Barcelona Center for International Health Research (CRESIB), Hospital Clinic-Universitat de Barcelona, Barcelona, Spain.; ^5^ Centro de Investigação em Saúde de Manhiça, Maputo, Mozambique.

**Keywords:** HIV/AIDS, malaria, antiretroviral drugs, pregnancy, mefloquine, mother-to-child transmission

## Abstract

Clinical trials demonstrated intermittent preventive treatment in pregnancy with mefloquine (MQ) reduced malaria rates among pregnant women, yet an unexpected higher risk of mother-to-child transmission (MTCT) of HIV among HIV-positive women receiving MQ has also been observed. To determine if interactions between antiretroviral drugs (ARVs) and MQ could contribute to the increased MTCT observed in women receiving MQ, we performed a retrospective cross-sectional analysis of ARV plasma concentrations in peripheral blood (maternal plasma) and cord blood (cord plasma) collected at delivery from 186 mothers participating in a randomized clinical trial of MQ (*n* = 102) compared with placebo (*n* = 84) in Kenya. Plasma zidovudine (AZT), lamivudine (3TC), and nevirapine (NVP) concentrations were measured by high-performance liquid chromatography–tandem mass spectrometry. Although only 4% (7/186) reported not using these ARVs, AZT, 3TC, and NVP were all below the limit of detection in 44% of maternal plasma and 42% of cord plasma samples, and proportions were similar between the two study arms. Median concentrations of AZT and 3TC were not significantly lower in the MQ arm compared with the placebo arm for maternal plasma and cord plasma (*p* > .05). However, median NVP concentrations were significantly lower in the MQ study arm compared with the placebo study arm in both maternal plasma (1,597 ng/mL vs. 2,353 ng/mL, Mann–Whitney Rank Sum, *p* = .023) and cord plasma (2,038 ng/mL vs. 2,434 ng/mL, *p* = .048). Reduced NVP concentrations in maternal and cord plasma of women receiving MQ suggest MQ may affect NVP metabolism for both mother and infant. These results highlight the need to evaluate potential drug–drug interactions between candidate antimalarials and ARVs for use in pregnant women.

Over 1 million pregnancies are complicated by coinfection with malaria and HIV, particularly in sub-Saharan Africa, where the public health burden of each pathogen is high.^1^ Antiretroviral therapy (ART) with combinations of antiretroviral medications (ARVs) has been effective in reducing mother-to-child transmission (MTCT) of HIV among pregnant women.^2^ Likewise, intermittent preventive treatment in pregnancy (IPTp) with antimalarial drugs effectively prevent malaria among HIV-negative women, yet potential drug–drug interactions between antimalarials and ARVs have not been fully explored among HIV-positive pregnant women. The antimalarial mefloquine (MQ) has a long half-life supporting once-monthly dosing, is considered safe in pregnancy, and has been shown to be highly effective against *Plasmodium falciparum* infection in Africa, making MQ an attractive candidate for IPTp in this region. Clinical trials have shown that IPTp with MQ effectively reduced rates of malaria and clinical complications among HIV-negative pregnant women.^3–5^ The Malaria in Pregnancy Preventive Alternative Drugs (MiPPAD) multicenter, double-blind, placebo-controlled clinical trial of MQ for IPTp also demonstrated the efficacy of MQ in preventing malaria among HIV-positive pregnant women. However, exploratory analyses from the MiPPAD trial revealed an unexpected higher risk of virological failure and MTCT of HIV among women receiving MQ compared with placebo^6^ raising concerns about potential interactions between MQ and ARV regimens that may have reduced antiretroviral efficacy for prevention of MTCT of HIV. To determine if interactions between ARVs and MQ exist and could contribute to the increased MTCT observed in women taking MQ, we performed a retrospective cross-sectional analysis of ARV concentrations at the time of delivery among pregnant HIV-positive Kenyan women participating in the MiPPAD trial.

We analyzed ARV concentrations in peripheral blood plasma (maternal plasma; 76 placebo study arm, 94 MQ study arm) and cord blood plasma (cord plasma; 74 placebo study arm, 88 MQ study arm) specimens collected from 186 MiPPAD study participants at the time of delivery at the Siaya District Hospital in Siaya, Kenya.^6^ HIV-positive mothers received directly observed MQ (Lariam; Roche, 250 mg/tablet) or placebo tablets at monthly antenatal clinic visits for a maximum of three doses and cotrimoxazole for daily prophylaxis. For this substudy, we analyzed all available specimens from mothers with virological failure (HIV viral load >1,000 copies/mL) at delivery as women with virological failure showed an increased risk of MTCT of HIV in the MiPPAD study.^6^ HIV/AIDS management was provided by local health services according to national HIV treatment guidelines at the time of the study^7^ and self-reported use of ARVs for either ART or prevention of MTCT was documented on study forms. Specimens were not selected based on specifically reported ARV regimens as ARVs were not directly provided as part of the intervention package in the MiPPAD study. Zidovudine (AZT), lamivudine (3TC), and nevirapine (NVP) were selected for analysis, as they were the ARVs reported to be used by >60% of women participating in the MiPPAD study.^6^ Plasma concentrations of AZT, 3TC, and NVP were measured in a single assay by high-performance liquid chromatography–tandem mass spectrometry with lower limits of quantification and detection of 20 ng/mL (AZT and 3TC) and 250 ng/mL (NVP).

The proportion of AZT, 3TC, or NVP detected in maternal plasma and cord plasma specimens was similar between the two study arms (Fig. 1). Overall, 4% (7/186) of mothers reported not using any of the three ARVs examined in this study; however, AZT, 3TC, and NVP were all below the limit of detection in 44% of maternal plasma and 42% of cord plasma specimens, which was similar between study arms. Combinations of AZT, 3TC, and NVP were also detected in maternal and cord plasma specimens in similar proportions between study arms (data not shown).

**FIG. 1.**
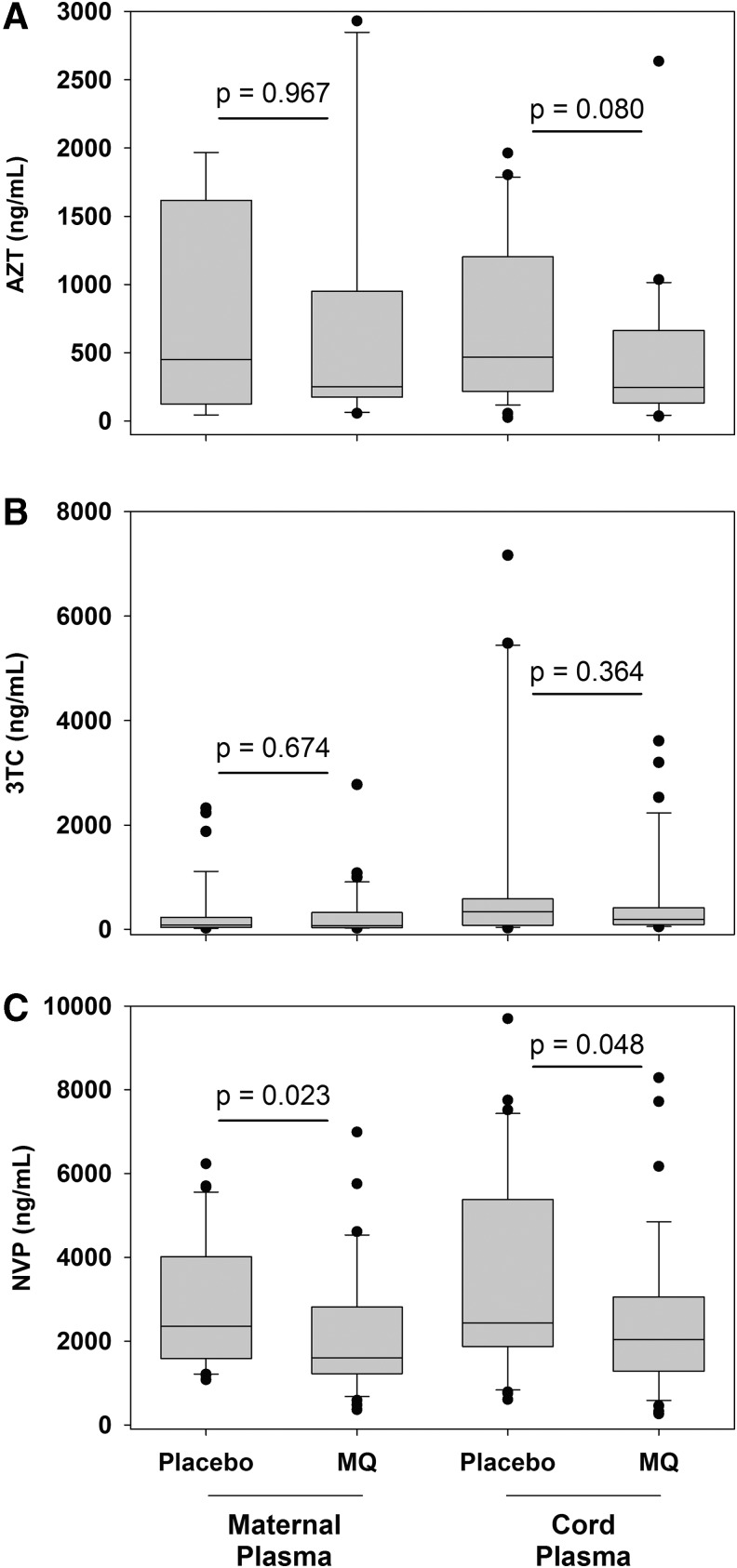
Maternal and cord plasma concentrations of zidovudine (AZT), lamivudine (3TC), and nevirapine (NVP) following intermittent preventive treatment with MQ or placebo for malaria control. Zidovudine (AZT) **(A)**, lamivudine (3TC), **(B)** and nevirapine (NVP) **(C)** concentrations are presented from maternal plasma (76 placebo study arm, 94 MQ study arm) and cord plasma (74 placebo study arm, 88 MQ study arm) specimens with detectable antiretroviral drug concentrations collected at delivery from HIV-infected pregnant women and their infants. AZT maternal plasma *n* = 9 (Control), 10 (MQ); cord plasma *n* = 26 (Control), 28 (MQ); 3TC maternal plasma *n* = 37 (Control), 39 (MQ); cord plasma *n* = 23 (Control), 31 (MQ); NVP maternal plasma *n* = 31 (Control), 34 (MQ); cord plasma *n* = 30 (Control), and 35 (MQ). MQ, mefloquine.

In specimens with detectable ARVs, median concentrations of AZT and 3TC were lower in the MQ arm compared with the placebo arm for maternal plasma and cord plasma, but these results were not statistically significant (*p* > .05) (Fig. 1A, B). Notably, median NVP concentrations were significantly lower in the MQ study arm compared with the placebo study arm in both maternal plasma (1,597 ng/mL vs. 2,353 ng/mL, Mann–Whitney Rank Sum, *p* = .023) and cord plasma (2,038 ng/mL vs. 2,434 ng/mL, *p* = .048) (Fig. 1C).

The results presented in this study provide the first information on potential drug–drug interactions between MQ and NVP that could play a role in the observed increase in MTCT of HIV among pregnant women who received MQ as IPTp. The proportions of women with detectable AZT, 3TC, and NVP were similar between study arms, but compared with the placebo study arm, NVP concentrations in both maternal and cord plasma were significantly reduced among participants in the MQ study arm. While it is unclear whether reduced NVP concentrations observed in this study in the presence of MQ decrease NVP efficacy, these NVP concentrations remain greater than those previously reported to prevent HIV replication.^8^ These observations suggest the reduction of NVP by MQ is not likely to be attributable solely to differences in ARV compliance between study arms. The 25% reduction of median NVP concentrations observed among women receiving MQ compared with placebo is similar to other known interactions of MQ with other drugs, including reduction of the ARV ritonavir and rifampicin for tuberculosis treatment.^9,10^ The potential for MQ and NVP to alter concentrations of each other has been previously speculated, namely because both drugs are metabolized by cytochrome P450 3A4 (CYP3A4),^11^ yet concerns have primarily focused on NVP reducing MQ efficacy rather than the converse.

This study has a number of limitations primarily because the MiPPAD trial was designed to assess effects of MQ on malaria infection among HIV-positive pregnant women, not to explore the effects of MQ on ARVs. Thus, longitudinal specimens throughout pregnancy and information such as the timing of previous ARV dosing were not collected, which might have been useful to identify ARV pharmacokinetic changes in the presence of MQ among study participants. However, randomization within the study is likely to limit the effect that timing of previous ARV dosing may have on the results presented in this study. Nevertheless, confirmation of potential interactions between MQ and NVP may require an independent *in vivo* pharmacokinetic assessment.

Identifying safe and effective treatment and prevention options for HIV and malaria among HIV-positive pregnant women is necessary to improve health outcomes for this vulnerable population in areas where the two diseases coexist. Multiple changes in absorption, disposition, metabolism, and excretion of drugs occur during pregnancy and evaluation of pharmacokinetic interactions should be appropriately evaluated.^12^ While NVP and MQ are unlikely to be used together as future clinical interventions, a better understanding of pharmacological interactions between newer ARV regimens and antimalarial drugs will be critical to determine if dose adjustments are needed to maintain efficacy of both drug classes. Such data collected through pharmacokinetic studies can provide valuable information for large clinical efficacy trials and reduce the likelihood of undesired outcomes due to unrecognized drug–drug interactions.
